# Free-Breathing 3D Liver Perfusion Quantification Using a Dual-Input Two-Compartment Model

**DOI:** 10.1038/s41598-017-17753-9

**Published:** 2017-12-13

**Authors:** Satyam Ghodasara, Shivani Pahwa, Sara Dastmalchian, Vikas Gulani, Yong Chen

**Affiliations:** Department of Radiology, Case Western Reserve University, and University Hospitals Cleveland Medical Center, Cleveland, Ohio, USA

## Abstract

The purpose of this study is to test the feasibility of applying a dual-input two-compartment liver perfusion model to patients with different pathologies. A total of 7 healthy subjects and 11 patients with focal liver lesions, including 6 patients with metastatic adenocarcinoma and 5 with hepatocellular carcinoma (HCC), were examined. Liver perfusion values were measured from both focal liver lesions and cirrhotic tissues (from the 5 HCC patients). Compared to results from volunteer livers, significantly higher arterial fraction, fractional volume of the interstitial space, and lower permeability-surface area product were observed for metastatic lesions, and significantly higher arterial fraction and lower vascular transit time were observed for HCCs (*P* < 0.05). Significantly lower arterial fraction and higher vascular transit time, fractional volume of the vascular space, and fractional volume of the interstitial space were observed for metastases in comparison to HCCs (*P* < 0.05). For cirrhotic livers, a significantly lower total perfusion, lower fractional volume of the vascular space, higher fractional volume of the interstitial space, and lower permeability-surface area product were noted in comparison to volunteer livers (*P* < 0.05). Our findings support the possibility of using this model with 3D free-breathing acquisitions for lesion and diffuse liver disease characterization.

## Introduction

Quantitative liver perfusion imaging using dynamic contrast-enhanced (DCE) MRI has been developed to investigate liver pathologies^1^. While a dual-input single-compartment model has been widely used^1–4^, it has been suggested that a more appropriate kinetic model for certain pathological settings such as cirrhosis and certain neoplasms requires two tissue compartments^5–7^. This is because of the complex microanatomy of liver tissues, which include two major spaces besides hepatocytes. One major space is the sinusoid system, which is a collection of small blood vessels containing mixed blood from the hepatic artery and the portal vein. This sinusoidal space can be considered the vascular space. Adjacent to the sinusoids is the Space of Disse, which can be considered the interstitial space^7^. Between the sinusoids and the Space of Disse are fenestrations, which allow for some degree of continuity between the two spaces. In healthy liver tissues, these fenestrations are open, allowing for approximately free communication so they effectively function as one space^3^. However, in certain liver pathologies, the fenestrations between the Space of Disse and the sinusoids close, making them function more as two independent spaces^3^.

Specifically, metastatic tumors in the liver are thought to be comprised of two independent spaces. These tumors are known to generate their own vasculature, which constitutes a vascular space^6^. The interstitium of these tumors are commonly desmoplastic, which constitutes a second, interstitial space^6^. In the case of liver cirrhosis, Ito cells deposit fibers, which enlarges the Space of Disse^7^. Additionally, more basement membrane is deposited, which closes off the fenestrae between the liver sinusoids and Space of Disse^7^. These microanatomic changes make flow between the sinusoids and Space of Disse in cirrhotic livers more difficult than flow in healthy livers, and so these two spaces behave more independently in cirrhotic livers^7^. Similar changes can also be seen in the setting of hepatocellular carcinoma (HCC)^8^. A majority of HCCs arise in a background of cirrhosis and as the dysplastic nodules progress to HCC, the sinusoids lose their fenestrae and become decreasingly porous^8,9^.

In view of these characteristics of various liver pathologies, the dual-input two-compartment model has recently been applied to patients with liver metastases and HCC^5,8,10,11^. However, most of these studies focused on application of this model to one type of lesion. Whether it can be used for differentiation of different types of lesions remains unknown. In addition, perfusion quantification using this model has not been applied to volunteer subjects to establish the baseline perfusion parameters for healthy liver parenchyma. The objective of this study is to test the feasibility of applying this model to DCE-MRI time series volumes of livers with a variety of focal lesions and compare these results to those obtained from volunteers using high spatiotemporal resolution free-breathing 3D acquisitions.

## Materials and Methods

### Dual-Input Two-Compartment Model for Liver Perfusion Quantification

The dual-input two-compartment model has been developed and described in depth by Koh, *et al*.^6^. Briefly, the contrast agent concentration-time curves in the liver tissue, hepatic artery, and portal vein are represented by ${C}_{t}(t)$, ${C}_{a}(t)$, and ${C}_{p}(t)$, respectively. Two delay parameters were included to account for the time delay between contrast agent arrival in the liver tissue and aorta and portal vein, respectively. Blood flow from the hepatic artery and the portal vein are represented by ${F}_{a}$ and ${F}_{p}$, respectively. $F$ represents the total blood flow to the liver and is equal to the sum of ${F}_{a}$ and ${F}_{p}$. $\alpha$ is the arterial fraction of the total blood flow to the liver and is equal to ${F}_{a}$/$F$. All of these quantities are related in the following formula:

1 $$\begin{array}{rcl}{C}_{t}(t) & = & [{F}_{a}{C}_{a}(t)+{F}_{p}{C}_{p}(t)]\ast R(t)\\  & = & F[\alpha {C}_{a}(t)+(1-\alpha ){C}_{p}(t)]\ast R(t)\end{array}$$

where * represents the convolution of the two functions. $R(t)$ is the tissue impulse residue function and is the sum of two functions, ${R}_{1}(t)$ (the vascular transit phase) and ${R}_{2}(t)$ (the parenchyma back flux phase), which are represented as:

2 $${R}_{1}(t)=u(t)-u(t-{t}_{1})$$

and

3 $${R}_{2}(t)=u(t)\{1-\exp (-\frac{PS}{F})\times [1+{\int }_{0}^{t}\exp (-\frac{PS}{{v}_{2}}\tau )\sqrt{\frac{PS}{{v}_{2}}\frac{PS}{F}\frac{1}{\tau }}{I}_{1}(2\sqrt{\frac{PS}{{v}_{2}}\frac{PS}{F}\tau })d\tau ]\}$$

$u(t)$ represents the Heaviside unit step function. ${t}_{1}$ represents the mean time for blood to flow through the vasculature. ${v}_{2}$ is the fractional volume of the interstitial compartment. $PS$ is the permeability-surface area product of the pathological tissue vasculature, and ${I}_{1}$ is the modified Bessel function of the first kind.

${R}_{1}(t)$, the vascular transit phase, is constant between time point 0 and time point ${t}_{1}$. This characteristic reflects the behavior of the injected tracer. The entire quantity of the injected tracer can be detected in the vascular space for a fixed amount of time, which is represented by ${t}_{1}$. As blood flows through the liver tissue, some blood plasma (along with the injected tracer) can be transferred to the interstitial space within ${t}_{1}$. The quantity of tracer that is transferred from the vascular space to the interstitial space is represented by the extraction ratio, $E$, in this equation:

4 $$E=1-exp(-PS/F)$$

After time point ${t}_{1}$, tracer that is left in the vascular space will be cleared through normal blood flow. However, the tracer previously transferred to the interstitial space will transfer back into the vascular space and will also be cleared by normal blood flow. This phenomenon is referred to as the parenchyma back flux phase, represented by ${R}_{2}(t)$. The behaviors of ${R}_{1}(t)$ and ${R}_{2}(t)$ both apply to an infinitely short bolus of contrast.

The dual-input two-compartment model is a distributed parameter model and makes assumptions about tracer kinetics and physiology. First, it assumes contrast agent concentration in the vascular space varies with time and the distance along the vessel, and second, it assumes contrast agent concentration in the interstitial space varies with time alone. This second assumption is dependent on contrast agent being well-mixed in the interstitial space after diffusing into the space.

### MRI Acquisitions

This study is HIPAA-compliant, and written informed consent was obtained from all subjects. The imaging protocol was approved by the University Hospitals Cleveland Medical Center Institutional Review Board and all the experiments were performed in accordance with the relevant guidelines and regulations. The dual-input two-compartment model was applied to 3D liver perfusion imaging data acquired from a Siemens 3 T Skyra scanner. 11 patients (M:F, 9:2; mean age 64.3 years with range 46–75 years) with focal liver lesions were scanned. Among them, 5 patients had HCC with 13 total lesions, and 6 patients had metastatic adenocarcinoma from lung, colon, or breast cancers with a total of 39 lesions. 7 healthy volunteers (M:F, 4:3; mean age 20.8 years with range 19–23 years) were also studied to establish the baseline perfusion values for healthy liver parenchyma.

For each subject, high spatiotemporal resolution 3D whole-liver dynamic contrast-enhanced (DCE) images were acquired using the through-time spiral GRAPPA technique. The details of this technique have been discussed in the literature^1,12^. A series of T_1_-weighted 3D liver images were acquired using a gradient echo sequence with a 3D stack-of-spirals trajectory. To meet the Nyquist criterion, 48 spirals were designed to fully cover the k-space. To achieve a high temporal resolution for accurate perfusion quantification, an in-plane reduction factor of 6 was applied and only 8 spiral interleaves were acquired for each 2D plane. The acquisition time for each 3D dataset with 60 partitions was 1.6–2.4 seconds. For each subject, a total of 100–120 volumes were acquired continuously in 3.2–4.5 minutes while the subject was breathing freely. A single dose (0.1 mmol/kg) of gadobenate dimeglumine (MultiHance; Braco Diagnostics, Princeton, NJ) was administered at 3 mL/s after the 5th volume was acquired, followed by 20 mL of saline solution. Other imaging parameters included: FOV, 38–46 cm; matrix size: 208 × 208 to 240 × 240 (with an effective in-plane resolution of about 1.9 mm); slice thickness: 3 mm; TR: 4.5–4.7 ms; TE: 0.5 ms; flip angle: 15°; partial Fourier in partition direction: 6/8. After the 4-minute dynamic scan was completed, a separate 40 second calibration scan with three fully-sampled volumes was also acquired during free-breathing to extract the GRAPPA weights for image reconstruction.

### Data Processing

The raw data were transferred offline to a local workstation for post-processing. The through-time spiral GRAPPA technique was first applied to reconstruct the undersampled DCE images using MATLAB (The Mathworks, Natick, MA). Similar to traditional Cartesian GRAPPA, a GRAPPA weight set was first calculated using the calibration scan. In the current study, a 2 × 3 GRAPPA kernel was used in the spiral arm × readout direction. Due to the non-Cartesian sampling pattern with the spiral trajectory, each GRAPPA kernel has a unique size and shape that is dependent on its own position in k-space. Therefore, specific GRAPPA weights were computed for each GRAPPA kernel and then applied to the undersampled dynamic data to fill the missing spiral interleaves. After the GRAPPA reconstruction, the k-space data were converted to anatomical images using a non-uniform Fast Fourier Transform (FFT) toolbox for gridding and image reconstruction^13^. Since the liver DCE images were acquired without any breath-holds, relative motion due to respiration exists between different volumes acquired at different time points. To obtain accurate perfusion quantification without motion contamination, a multi-reference image registration step was applied after image reconstruction using the FMRIB’s Non-linear Image Registration Tool (FNIRT)^14^.

After all the images were co-registered, the dual-input two-compartment model was applied on a voxel-by-voxel basis for liver perfusion quantification. This computation was performed using the non-linear curve fitting function in MATLAB. The integrals included in calculating the convolution and ${R}_{2}(t)$ in the dual-input two-compartment model were estimated by the rectangular method. The width, $dt$, of each rectangle was assumed to be equal to the temporal resolution of the acquisition (1.6–2.4 seconds). Regions-of-interest (ROIs) were drawn on multiple slices to measure arterial and portal input functions for each subject. Six perfusion parameters, including total blood flow ($F$), arterial fraction ($\alpha$), vascular transit time (${t}_{1}$), fractional volume of the vascular space (${v}_{1}$), fractional volume of the interstitial space (${v}_{2}$), and permeability-surface area product ($PS$) were quantified. To extract perfusion values from patients with focal liver lesions, ROI analysis was performed by a radiologist with eight years of experience. For the HCC patients, ROIs were also drawn in liver parenchyma outside of the focal lesions to extract perfusion information from cirrhotic tissue. All the perfusion numbers were compared to the values obtained from volunteers.

### Statistical analyses

A two-tailed Student’s $t$-test was used to compare the perfusion values between two groups. A $P$-value less than 0.05 was deemed to be significantly different.

### Data availability

The datasets analysed during the current study are available from the corresponding author on reasonable request.

## Results

**Figure 1:**
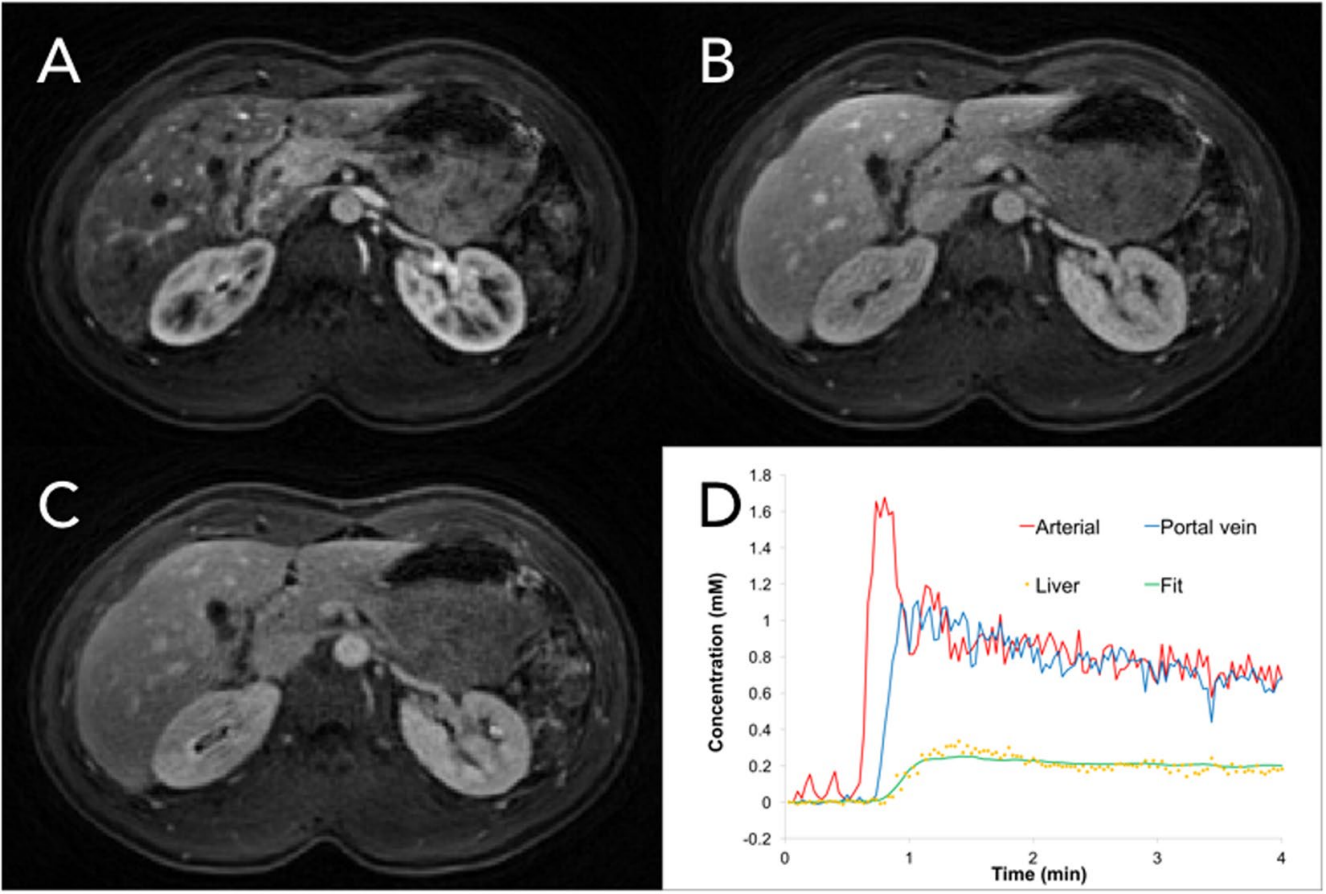
(**A**–**C**) Representative liver DCE images obtained from a volunteer in the arterial phase, portal phase, and delayed phase. (**D**) Representative time-course curves of contrast concentration from the aorta, portal vein, and single-voxel liver tissue. The fitted curve of liver data using the dual-input two-compartment model was also plotted (green line). The fitting yielded a total blood flow ($F$) of 81.0 mL/min/100 mL, arterial fraction ($\alpha$) of 31.0%, vascular transit time (${t}_{1}$) of 23.3 s, fractional volume of the vascular space (${v}_{1}$) of 18.9%, fractional volume of the interstitial space (${v}_{2}$) of 5.7%, and permeability-surface area product ($PS$) of 35.1 mL/min/100 mL.

**Figure 2:**
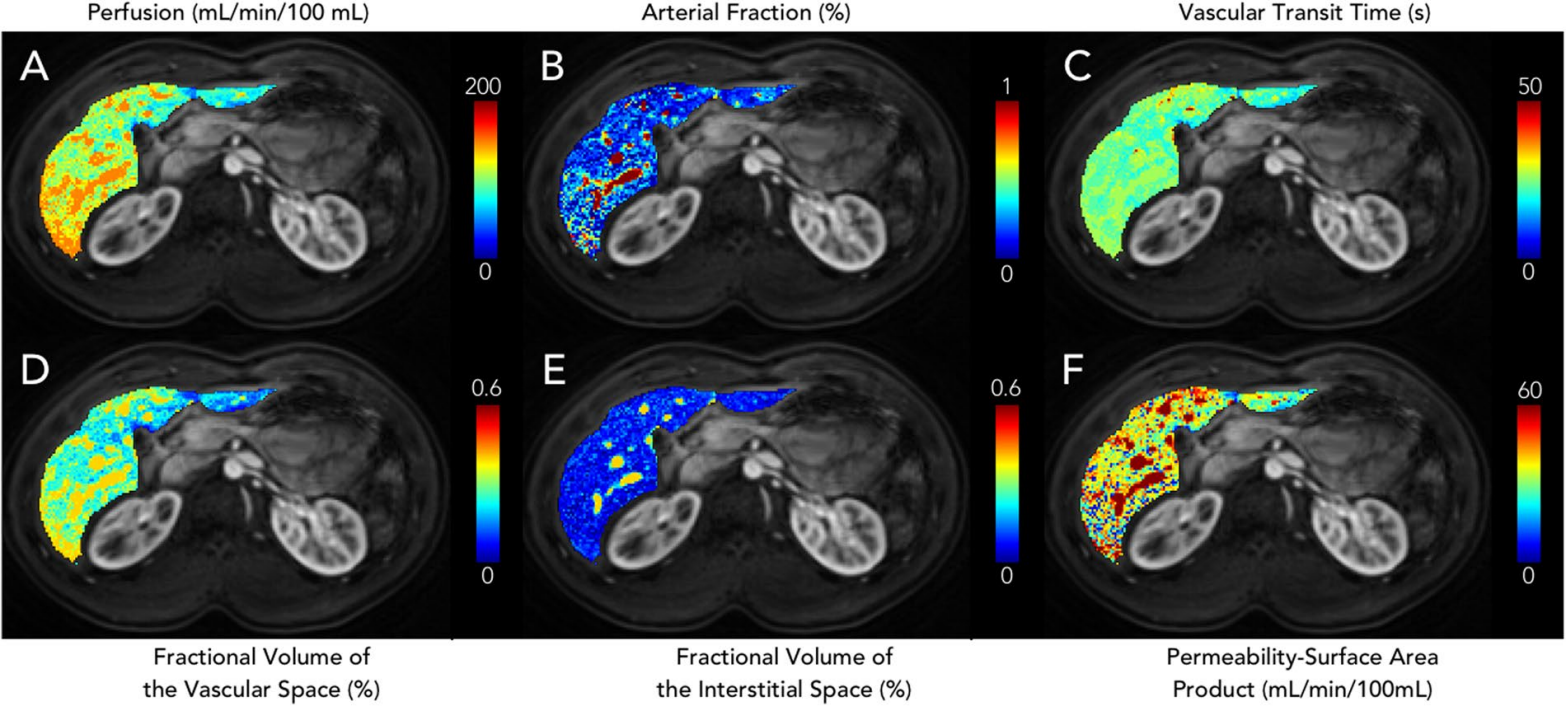
Representative liver perfusion maps from a volunteer. (**A**) Total blood flow ($F$), (**B**) arterial fraction ($\alpha$), (**C**) vascular transit time (${t}_{1}$), (**D**) fractional volume of the vascular space (${v}_{1}$), (**E**) fractional volume of the interstitial space (${v}_{2}$), and (**F**) permeability-surface area product ($PS$).

Figure 1 shows representative liver DCE images and time-course curves of contrast concentration obtained from a volunteer. Three contrast concentration curves, including one voxel from the aorta, one voxel from the portal vein, and one voxel from the liver tissue, are plotted (Fig. 1D). For volunteer liver tissue, the contrast concentration reached peak value around one minute and then gradually decreased during washout. Fitted data to the liver tissue using the dual-input two-compartment model are also presented. Figure 2 shows corresponding perfusion maps obtained from the same volunteer. For liver parenchyma from this subject, the average fractional volume of the vascular space (${v}_{1}$) was 31.6 ± 6.8% and the fractional volume of the interstitial space (${v}_{2}$) was 10.0 ± 4.9%.

**Figure 3:**
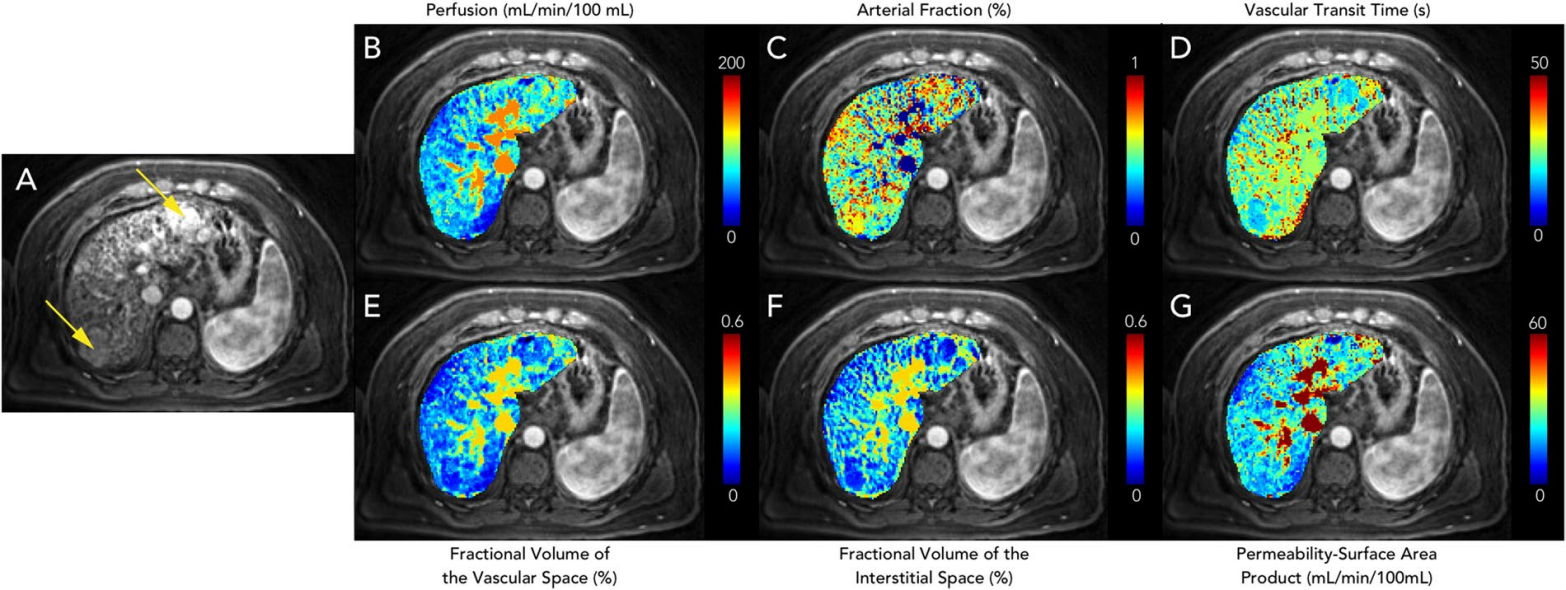
Representative liver perfusion maps from a patient with two HCC lesions. (**A**) An arterial phase T_1_-weighted image acquired with the free-breathing liver perfusion imaging technique. (**B**–**G**) Corresponding perfusion maps for various parameters.

**Figure 4:**
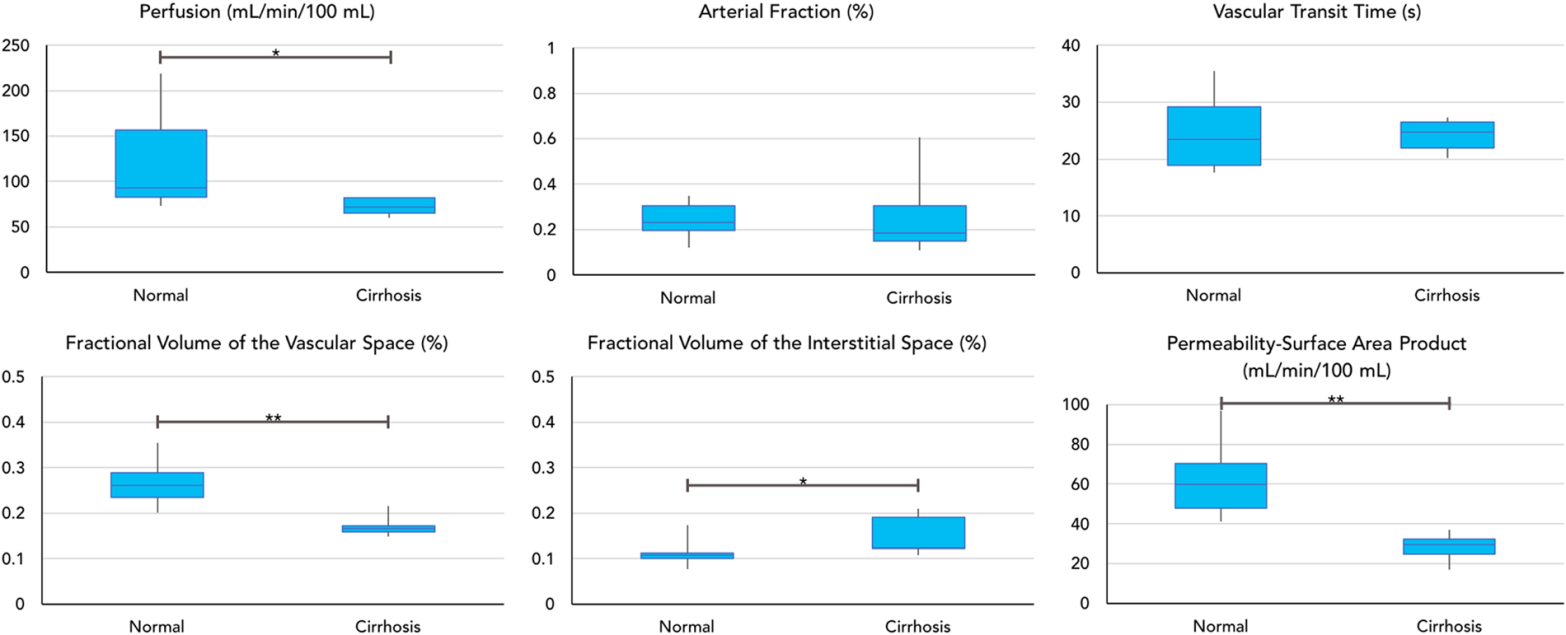
Summary of perfusion parameters of liver tissue from volunteers ($n=7$) and cirrhotic tissues in HCC patients ($n=5$). *$P < 0.05$ between the two groups; **$P < 0.01$ between the two groups.

Figure 3 shows representative perfusion maps from a patient with HCC. Two lesions with size of 38.0 mm and 34.2 mm were noted for this subject (Fig. 3A). Compared to surrounding liver tissue, a higher arterial fraction was observed for both lesions (Fig. 3C), while the vascular transit time and fractional volume of the interstitial space were lower (Fig. 3D & F). Comparison of the perfusion values was further performed between volunteer liver tissues ($n=7$) and cirrhotic tissues in HCC patients ($n=5$), and the results are summarized in Fig. 4. Compared to the volunteer liver parenchyma, the total perfusion ($F$) was significantly decreased in cirrhosis (123.6 ± 55.7 mL/min/100 mL vs. 70.9 ± 9.5 mL/min/100 mL; $P < 0.05$), the fractional volume of the vascular space (${v}_{1}$) was significantly decreased (26.6 ± 5.5% vs. 17.6 ± 2.5%; $P < 0.01$), the fractional volume of the interstitial space (${v}_{2}$) was significantly increased (11.2 ± 3.0% vs. 16.6 ± 5.6%; $P < 0.05$), and the permeability-surface area product ($PS$) was significantly decreased (62.1 ± 19.6 mL/min/100 mL vs. 27.8 ± 6.9 mL/min/100 mL; $P < 0.01$). For volunteer liver tissue, the fractional volume of the vascular space (${v}_{1}$) was significantly higher than the fractional volume of the interstitial space (${v}_{2}$; $P < 0.0001$), which is consistent with the findings shown in Fig. 2DE. No statistical difference was noted in cirrhosis between these two fractional volumes ($P > 0.05$).

**Figure 5:**
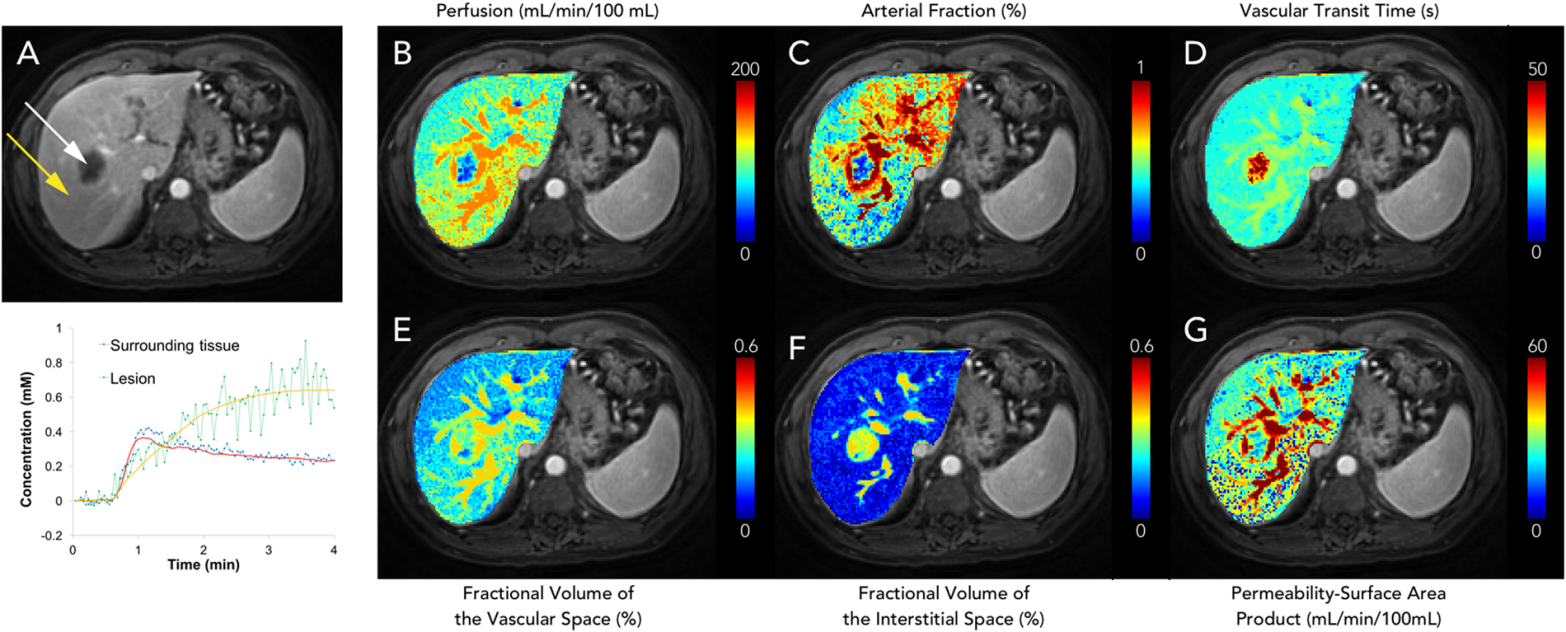
Representative liver perfusion maps from a patient with metastatic lung adenocarcinoma. (**A**) A T_1_-weighted image and single-voxel contrast concentration curves from both the lesion (white arrow) and surrounding liver tissue (yellow arrow). Fitted data using the dual-input two-compartment model were also plotted. (**B**–**G**) Corresponding perfusion maps for various parameters.

The dual-input two-compartment model was further applied to patients with metastatic adenocarcinoma lesions. Figure 5A shows representative contrast concentration curves for two voxels over time for a patient with liver metastases from lung adenocarcinoma. Two contrast concentration curves from single-voxel measurements were plotted: one for the lesion itself and one for the tissue surrounding the lesion. The fitted curves using the dual-input two-compartment model were also plotted. While a similar enhancement curve was observed in the surrounding tissue as compared to volunteer liver tissue (Fig. 1D), a distinct and gradually increasing enhancement curve was noticed for the metastatic lesion. The corresponding perfusion maps of all parameters for this patient are presented in Fig. 5B–G. Marked increases in both vascular transit time (${t}_{1}$) and the fractional volume of the interstitial space (${v}_{2}$) were noted in the lesion as compared to the surrounding liver parenchyma (Fig. 5D,F). One pattern to note is that for metastatic patients, the fractional volume of the interstitial space (${v}_{2}$) in the surrounding tissue is evidently lower than the fractional volume of the vascular space (${v}_{1}$), which is similar to the pattern observed for volunteer liver parenchyma (Fig. 2D,E).

**Figure 6:**
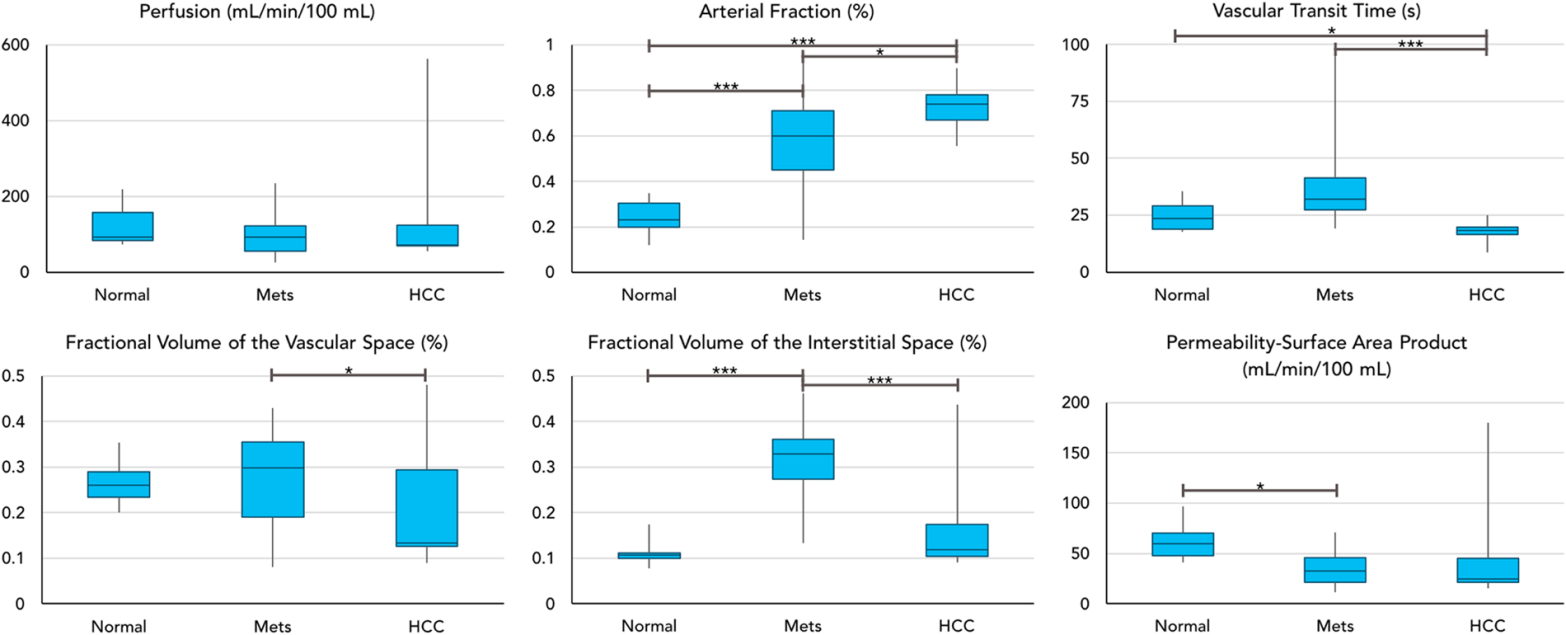
Summary of perfusion parameters from focal liver lesions and the comparison to results from volunteer liver tissues. *$P < 0.05$ between the two groups; ***$P < 0.001$ between the two groups.

A summary of all the perfusion parameters for both HCC and liver metastases are presented in Fig. 6, and the results are compared to those obtained from volunteers. Significant differences were observed in the arterial fraction ($\alpha$) among the three groups ($P < 0.05$). Both HCC and liver metastases had a significantly higher arterial fraction as compared to that of volunteers (volunteers, 24.4 ± 8.0%; metastases, 58.5 ± 20.1%; HCC, 73.2 ± 9.1%; $P < 0.05$). The arterial fraction in HCC was also significantly higher than that of liver metastases ($P < 0.05$). HCC also had a significantly lower vascular transit time than either volunteer liver tissue or metastatic tissues (volunteers, 24.7 ± 6.8 sec; metastases, 36.3 ± 15.7 sec; HCC, 17.8 ± 4.7 sec; $P < 0.05$). On the other hand, liver metastases had a significantly higher fractional volume of the vascular space (${v}_{1}$) than HCC (metastases, 28.1 ± 10.0%; HCC, 21.1 ± 12.6%; $P < 0.05$) and a significantly higher fractional volume of the interstitial space (${v}_{2}$) than both of the other groups (volunteers, 11.2 ± 3.0%; metastases, 31.6 ± 7.3%; HCC, 16.8 ± 10.6%; $P < 0.001$ as compared to HCC and volunteer liver tissue).

## Discussion

This study shows initial feasibility of using a dual-input two-compartment model in 3D free-breathing lesion characterization. Many studies have shown that quantitative liver perfusion imaging can provide valuable information to characterize lesions, predict treatment response, and monitor therapeutic effects^5,6,10,15,16^. Most studies to date were performed using CT imaging due wider availability and fast image acquisition. However, CT is limited by the use of ionizing radiation, especially for this type of dynamic measurement. In the past twenty years, MRI acquisition speed has improved dramatically, and multiple technologies have been developed for dynamic liver imaging^1,17,18^. Accurate and robust perfusion quantification using these imaging data with appropriate tracer kinetic models is one of the key steps to make MRI more applicable for liver perfusion imaging.

The perfusion results obtained for both focal liver lesions and cirrhosis in the current study are in agreement with literature findings obtained using the dual-input two-compartment model or other analytical models. For example, Van Beers *et al*. measured liver perfusion from 18 patients with cirrhosis using dynamic CT. They reported an average total blood flow ($F$) of 67 ± 23 mL/min/100 mL and arterial fraction ($\alpha$) of 41 ± 27% using a dual-input single-compartment model, which match the findings reported here for cirrhotic tissue^19^. Koh *et al*. also investigated cirrhotic tissues using dynamic contrast-enhanced CT coupled with the same dual-input two-compartment model^8^. For the four patients included in that study, the fractional volume of the vascular space (${v}_{1}$) is in a range of 16.0 to 22.2%, and the fractional volume of the interstitial space (${v}_{2}$) is between 10.7 and 42.2%, which are also in good agreement with our results for cirrhosis (${v}_{1}$, 17.6 ± 2.5%; ${v}_{2}$, 16.6 ± 5.6%). For HCC in this study, the arterial fraction ($\alpha$) was significantly higher and the vascular transit time (${t}_{1}$) was significantly lower compared to volunteer liver tissue. These findings are consistent with early CT studies and the expectation for HCC, which enhances at an early arterial phase^20,21^. Several other studies have also examined hepatic metastases using the dual-input two-compartment model. Koh *et al*. have applied this model to dynamic contrast-enhanced MRI data from three patients^5^. They found a higher fractional volume of the interstitial space (${v}_{2}$) for metastatic lesions compared to the surrounding tissue, which is also consistent with our findings.

Besides diseased liver tissues, we also investigated liver perfusion in volunteers. The fractional volume of the interstitial space (${v}_{2}$) was found to be significantly lower than the fractional volume of the vascular space (${v}_{1}$) in volunteer liver parenchyma, in keeping with the fact that open fenestrations in healthy livers allow free communication between the liver sinusoids and the Space of Disse. This is further confirmed by a significantly higher permeability-surface area product ($PS$) in volunteer liver tissue compared to cirrhotic liver tissues. While all these findings indicate that a dual-input single-compartment model can be applied to volunteer liver tissues, the findings from volunteer liver tissues obtained by using the dual-input two-compartment model can be directly compared to the findings obtained from abnormal liver tissues to help identify changes in specific physiological properties leading to disease.

In the current study, we also compared liver perfusion values obtained from two different types of focal lesions: liver metastases and HCC. Significant differences were noted in the arterial fraction ($\alpha$), vascular transit time (${t}_{1}$), the fractional volume of the vascular space (${v}_{1}$), and the fractional volume of the interstitial space (${v}_{2}$). Compared to metastatic adenocarcinoma, HCC is a highly vascular solid tumor, and tumoral neoangiogenesis from the hepatic artery plays a crucial role in disease progression. This leads to a more dominantly arterial blood supply, which in turn leads to a significantly higher arterial fraction ($\alpha$). Most HCC lesions exhibit early arterial phase enhancement and fast washout, while liver metastases typically show slow enhancement. This supports the significant differences observed in the vascular transit times (${t}_{1}$) between the two types of lesions in this study. The significantly lower fractional volume of the interstitial space in HCC compared to metastatic lesions has not been reported before. While the underlying mechanism is unknown, it is likely due to an increase in fibrotic tissues within the interstitial space for HCC lesions. Further studies are needed to test this hypothesis.

There are some limitations to this study. First, the number of patients is small in this initial pilot study. Additionally, the age ranges between the volunteer and patient groups are substantially different (volunteers, 19–23 years; patients, 46–75 years). Data obtained from the younger volunteers may not apply to the older patients. There is large variability in some perfusion parameters, such as the fractional volume of the vascular space (${v}_{1}$) and permeability-surface area product ($PS$) for HCC lesions. Second, the liver metastases reported in the current study, while all adenocarcinoma, included lesions from different primary tumors and there may be differences in perfusion characteristics between lesions from different source organs^22^. More patient data are needed to perform this type of comparison using the dual-input two-compartment model.

In conclusion, a dual-input two-compartment model was applied to 3D free-breathing liver perfusion data acquired from both volunteers and patients with different lesions. Significant differences in perfusion parameters were found between volunteer liver parenchyma, cirrhotic liver, and focal liver lesions as HCC and metastases. The dual-input two-compartment model with 3D free-breathing acquisitions could potentially be applied for quantitative characterization and follow-up of focal liver lesions.

## References

[1] Chen Y (2015). Free-breathing liver perfusion imaging using 3D through-time spiral GRAPPA acceleration. Invest. Radiol..

[2] Materne R (2002). Assessment of hepatic perfusion parameters with dynamic MRI. Magn. Reson. Med..

[3] Kim SH, Kamaya ACT (2014). Perfusion of theLiver: Principles and Applications in. Radiology.

[4] Bultman EM (2014). Quantitative hepatic perfusion modeling using DCE-MRI with sequential breathholds. J. Magn. Reson. Imaging.

[5] Koh TS (2008). Hepatic Metastases: *In Vivo* Assessment of Perfusion Parameters at Dynamic Contrast-enhanced MR Imaging with Dual-Input Two-Compartment Tracer Kinetics Model. Radiology.

[6] Koh TS (2011). Dynamic contrast-enhanced MRI of neuroendocrine hepatic metastases: A feasibility study using a dual-input two-compartment model. Magn. Reson. Med..

[7] Thng CH (2010). Perfusion magnetic resonance imaging of the liver. World J. Gastroenterol..

[8] Koh TS (2009). Dynamic contrast-enhanced CT imaging of hepatocellular carcinoma in cirrhosis: Feasibility of a prolonged dual-phase imaging protocol with tracer kinetics modeling. Eur. Radiol..

[9] Sanyal AJ, Yoon SK, Lencioni R (2010). The etiology of hepatocellular carcinoma and consequences for treatment. Oncologist.

[10] Sourbron S, Reiser MF, Zech CJ (2012). Combined quantification of liver perfusion and function with dynamic gadoxetic acid – enhanced MR imaging. Radiology.

[11] Yang J-F (2016). Dual-input two-compartment pharmacokinetic model of dynamic contrast-enhanced magnetic resonance imaging in hepatocellular carcinoma. World J. Gastroenterol..

[12] Seiberlich N (2011). Improved temporal resolution in cardiac imaging using through-time spiral GRAPPA. Magn. Reson. Med..

[13] Fessler JA (2007). On NUFFT-based gridding for non-Cartesian MRI. J. Magn. Reson..

[14] Jenkinson M, Beckmann CF, Behrens TEJ, Woolrich MW, Smith SM (2012). FSL. Neuroimage.

[15] Hagiwara M (2008). Advanced liver fibrosis: diagnosis with 3D whole-liver perfusion MR imaging–initial experience. Radiology.

[16] Miyazaki K (2012). Neuroendocrine tumor liver metastases: use of dynamic contrast-enhanced MR imaging to monitor and predict radiolabeled octreotide therapy response. Radiology.

[17] Zhang T (2015). Fast pediatric 3D free-breathing abdominal dynamic contrast enhanced MRI with high spatiotemporal resolution. J. Magn. Reson. Imaging.

[18] Feng L (2014). Golden-angle radial sparse parallel MRI: combination of compressed sensing, parallel imaging, and golden-angle radial sampling for fast and flexible dynamic volumetric MRI. Magn. Reson. Med..

[19] Van Beers BE (2001). Hepatic Perfusion Parameters in Chronic Liver Disease. Am. J. Roentgenol..

[20] Sahani DV, Holalkere N-S, Mueller PR, Zhu AX (2007). Advanced hepatocellular carcinoma: CT perfusion of liver and tumor tissue–initial experience. Radiology.

[21] Zhu AX, Holalkere NS, Muzikansky A, Horgan K, Sahani DV (2008). Early antiangiogenic activity of bevacizumab evaluated by computed tomography perfusion scan in patients with advanced hepatocellular carcinoma. Oncologist.

[22] Tsushima, Y., Funabasama, S., Aoki, J., Sanada, S. & Endo, K. Quantitative Perfusion Map of Malignant Liver Tumors, Created from Dynamic Computed Tomography Data. *Acad*. *Radiol*. 10.1016/S1076-6332(03)00578-6 (2004). 10.1016/s1076-6332(03)00578-6 14974597

